# The role of the junctional zone in the management of adenomyosis with infertility

**DOI:** 10.3389/fendo.2023.1246819

**Published:** 2023-10-10

**Authors:** Sha Wang, Hua Duan

**Affiliations:** Department of Minimally Invasive Gynecology, Beijing Obstetrics and Gynecology Hospital, Capital Medical University, Beijing Maternal and Child Health Care Hospital, Beijing, China

**Keywords:** adenomyosis, junctional zone, infertility, diagnosis, therapy

## Abstract

The junctional zone (JZ) is an important structure in the myometrium that maintains uterine fertility. Changes in the junctional zone are closely related to infertility and adenomyosis (ADS). As an increasing number of young women are affected by ADS, the disease is no longer considered typical of women over 40. With these changes, an increasing number of patients refuse hysterectomy and desire fertility preservation treatment. At the same time, ADS is a crucial factor causing female infertility. Therefore, the treatment of ADS-related infertility and preservation of reproductive function is one of the other major challenges facing clinicians. For these young patients, preserving fertility and even promoting reproduction has become a new challenge. Therefore, we searched and summarized these studies on PubMed and Google Scholar using keywords such as “adenomyosis”, “junctional zone”, and “infertility” to explore infertility causes, diagnosis, and treatment of ADS patients who wish to preserve their uterus or fertility and become pregnant, focusing on the junctional zone, to obtain a full appreciation of the new perspective on this disease.

## 1 Introduction

Adenomyosis is defined as the benign invasion of the endometrium into the myometrium, which microscopically exhibits endometrial glands and stroma surrounded by the hypertrophic and hyperplastic myometrium (1). It is characterized by abnormal uterine bleeding (AUB), pelvic pain symptoms, and infertility. However, the clinical presentation of adenomyosis is often mixed but can occasionally be asymptomatic (2). The disease is no longer considered typical of women over 40 years of age, as an increasing number of young women are affected by adenomyosis. Moreover, adenomyosis is diagnosed in 22% of infertile women under 40 years old undergoing treatment by assisted reproductive technology (ART) (3).

In 1983, shortly after magnetic resonance imaging (MRI) became available, the junctional zone (JZ) was described initially as a low-intensity band between the endometrium and the myometrium. The junctional zone is a distinct, hormone-dependent endometrial-myometrial interface, with a Müllerian origin, and the thickness changes during the menstrual cycle (4). In the nonpregnant uterus, highly specialized contraction waves originate exclusively from the JZ and participate in regulating diverse reproductive events, such as sperm transport, embryo implantation, and menstrual shedding (5).

Abnormal widening of the JZ is the consequence of uncoordinated inner myocyte proliferation called JZ hyperplasia (6). JZ hyperplasia and accompanying disruption could initiate endometrial mucosal penetration of endometrial glands into the myometrium (7). Alterations in JZ thickness and invasion of the endometrium into the inner myometrium could represent an early stage in the development of adenomyosis (8). The role of JZ in normal physiology and fertility suggests that abnormalities in this zone can cause disease and complications. Moreover, regular cyclical changes in myocytes in women with adenomyosis are absent, which may lead to many changes in ultrastructure (9).

Therefore, the focus on the JZ represents a new direction for patients with adenomyosis who wish to preserve or restore their fertility. Moreover, diagnosis and treatment of the JZ may be a solution to the plight of young women with adenomyosis. Previous articles have reviewed the function of the JZ in infertility (10, 11), but this article updates this part of the content of the latest literature and reviews the pathophysiological mechanism of JZ infertility, the role of imaging in diagnosis, and the influence of conservative treatment on infertility in adenomyosis. Impact of junctional zone on infertility.

The JZ consists of a three-dimensional (3D) mesh of irregular, mainly circular, and short muscular bundles and is part of the “archimetra” or “old uterus” seen in most vertebrate mammals. The two outer layers of the myometrium, the “neometra,” developed later in the evolutionary process to support childbirth, primarily due to the imbalance between the narrow pelvis and the fetal head (12). JZ contractility provides the main contractility of the unpregnant uterus. It plays a role in the fertility and pathogenesis of certain uterine diseases and symptoms through a hormone-dependent pattern. In the proliferative phase, retrograde contraction from the cervix to the fundus, and in the secretory phase, antegrade contraction from the fundus to the cervix play a role in sperm transport and early pregnancy preservation, respectively (5). The role of the JZ in normal physiology and fertility suggests that abnormalities in this zone can cause disease and complications. The structure and function of the JZ are lost during implantation and endometrial diseases, which can easily lead to the infiltration of trophoblasts or endometrial fragments in adenomyosis. Radiographic JZ thickening is a negative predictor of embryo implantation after IVF. Microenvironmental changes, such as abnormal contractions, hormonal changes, and immune abnormalities, can adversely affect the reproductive process and contribute particularly to excessive JZ peristalsis, which may lead to the development of uterine disease (13).

### 1.1 Effects of abnormal JZ thickness on fertility

The average thickness of the JZ has no clear standard. In early studies, the thickness of the JZ was defined as 5 mm to 8 mm. The thickness of the JZ increases in patients with adenomyosis, and the development of MRI technology provides the possibility for accurate measurement of JZ thickness. JZ MRI showed that a maximum thickness of the JZ ≥12 mm is a diagnostic criterion for adenomyosis (14). Currently, there is substantial research evidence indicating that increased JZ thickness can have adverse effects on reproduction and that there is a strong association between adenomyosis and reproductive impairment in women with a thickened JZ. Increased JZ thickness is considered a poor prognostic factor for implantation and is significantly associated with IVF implant failure. The average JZ is >7 mm, the maximal JZ is >10 mm, and the implantation failure rate is 95.8%. The IVF patient results show more than 74% failure. JZ thickening is an independent factor for embryo implantation failure, which is primarily unrelated to embryo quality or infertility subtype (15). Research has shown that the thinner the junctional area on the day of ovum pick up (OPU) is, the higher the implantation rate will be and that pregnant patients have a lower JZ thickness than nonpregnant patients, with an average JZ thickness of 0.27 cm (16). Compared with that of intrinsic type adenomyosis, the JZ area of adenomyosis in the extrinsic type is almost unchanged. At the same time, the extrinsic type shows a higher live birth rate and lower abortion rate. As the disease progresses, the miscarriage rate increases in the third trimester, which may be due to the involvement of impaired and deep placentation (14). In summary, JZ thickening is an important factor in reproductive disorders.

### 1.2 Effect of abnormal JZ contraction on fertility

Uterine peristalsis is a vital biomechanical activity for reproduction and fertility, and its dysfunction has a widespread impact on adenomyosis. Uterine peristalsis is spontaneous and coordinated contraction and relaxation in the nonpregnant uterus. JZ contraction varies in different periods, showing contraction from the cervix to the fundus in the nonpregnant period and contraction from the fundus to the cervix during pregnancy. Abnormal uterine contraction may be the basis of important diseases such as dysmenorrhea, infertility, endometriosis, implantation failure, spontaneous inflammation, abortion, and premature babies (17). Based on the various stages of the menstrual cycle, many studies have shown changes in the magnitude, frequency, and direction of contractions in the uterine JZ of normal uterus and ADS (18). Spontaneous peristalsis of the myometrium in the nonpregnant uterus plays a vital role in human reproduction. It involves menstrual blood discharge, sperm transport, and embryo implantation (19). Therefore, the JZ plays an essential role in normal reproduction, as shown in **Table 1**. There have been many related studies on the impact of structural and functional changes in the JZ on infertility management. The evolution of JZ contractility and infertility treatment can affect each other, often leading to poor treatment outcomes. In adenomyosis, the contraction of the JZ is disordered and irregular, and the enhancement of JZ activity will affect embryo implantation and sperm transportation during natural conception and assisted reproduction. Directional uterine contractility and integrity of uterine and fallopian tube transport function are necessary for sperm transport (24). Higher JZ activity and a corresponding increase in endometrial mobility may impair uterine receptivity and affect implantation. Contraction of the junctional area (JZ) during embryo transfer is associated with adverse outcomes, and factors that increase JZ contraction should be avoided. MRI shows interference of the sperm transport process by the excessive uterine peristalsis in adenomyosis, occurring mainly when diffuse adenomyosis affects the whole myometrium, and the sperm transport capacity is completely lost. Adenomyosis destroys the functional structure of the myometrium, which is responsible for directional sperm transport, thus affecting sperm transport and reducing the natural pregnancy rate (25). The study by Lesny P et al (22) evaluated the contraction of JZ during IVF and embryo transfer cycles in oocyte donors exposed to long-term ovarian stimulation protocols. When downregulated, no JZ shrinkage was observed. Seven days after superovulation, all patients displayed cervico-fundal, fundo-cervical, and random contractions. When injected with human chorionic gonadotropin-gonadotropin, the cervico-fundal waves dominated the image. However, the activity was most potent on the day of oocyte retrieval. All patients had JZ activity on the 2nd, 3rd, and 4th days after oocyte retrieval. Nevertheless, the regular wavy contractility gradually decreased, and only a single random movement was observed on the 4th day after oocyte retrieval. In summary, JZ activity was more significant throughout the IVF cycle than in the natural cycle. In another study by Biervliet FP et al. (23), patients with previous complex embryo transfers or complex mock embryo transfers received a transmyometrial embryo transfer (TMET). They found that TMET is a potent stimulus for JZ contractility and that increased contraction of the JZ results in abnormal implantation of embryos and ectopic pregnancy. In contrast, JZ contraction in the embryo transfer area is associated with adverse outcomes, and factors that increase JZ should be avoided. Lower endometrial activity during pregnancy is related to a higher pregnancy rate. The frequency of uterine peristalsis waves before embryo transfer negatively correlates with clinical pregnancy in fresh and frozen-thawed embryo transfer cycles. Compared with patients with a higher frequency, patients with uterine peristalsis waves < 3.0 waves/min before embryo transfer have higher chances of a successful pregnancy (26). Abnormal contraction of the JZ will also cause uterine pressure in the uterine cavity to rise, which will affect pregnancy. Smooth muscle contraction produces high and periodic intrauterine pressure on embryos, which is produced by uterine smooth muscle contraction and has the highest and most frequent occasional peak after implantation. Studies have shown that uterine smooth muscle contractions produce high and irregular intrauterine pressure. Improper contraction of uterine muscles leads to premature or delayed delivery (27).

**Table 1:** Effects of JZ changes in infertility management.

Authors	Study type	Results	Conclusions
**Maged et al., 2017 (** 16)	Prospective	In fundal, anterior, and posterior sites of the uterus, the difference in JZ measurements between women with ICSI success and those with ICSI failure was significant.	The thinner the junctional zone on the OUP day is, the higher the embryo sac implanting rate. Moreover, the difference in JZ thickness between the downregulation day and the OUP day is an excellent prediction of the outcome of the ICSI cycle.
**Maubon et al., 2010 (** 15)	Prospective	The increase in junctional zone thickness was significantly associated with IVF failure. Patients with an average junctional zone exceeding 7 mm and a maximum junctional zone exceeding 10 mm had an implantation failure rate of 95.8%. The implant failure rate in the other patient groups was 37.5%. The cause of infertility or the age of the patient has nothing to do with this difference in failure rates.	In a group of infertile patients undergoing IVF, a pelvic MRI scan showing a thickened uterine JZ was a negative predictor of embryo implantation after IVF.
**Liesbeth J. Meylaerts et al., 2017 (** 20)	Respective	Compared with the control group, the JZ at the posterior wall of the isthmus in women with anovulatory was significantly thicker. The infertile women receiving ovarian stimulation had a significantly thicker outer myometrium at the anterior wall.	The results showed that JZ thickening, especially thickening of the outer myometrium, may be related to infertility.
**Kunz & Beil, 2010 (** 21)	Prospective	In all women with metaphase I (MI)/germinal vesicle (GV) = 0%, compared with women with MI/GV> 0%, the JZ significantly increased and the clinical pregnancy rate decreased significantly. Infant take-home rates tend to be the same, but the differences are not significant. No direct effect of JZ on all other parameters can be observed.	The JZ recorded on MRI may interfere with follicular function.
**Lesny et al., 1998 (** 22)	Prospective	The highest wave frequency and velocity of JZ contraction were observed when the oocytes were retrieved (4.29 ± 0.68 waves/minute and 2.73 ± 0.54 mm/s, respectively).	Compared to the results observed in the natural cycle, JZ activity throughout the IVF cycle is more exaggerated, although following a similar pattern.
**Biervliet et al., 2002 (** 23)	Prospective	The effect of transmyometrial embryo transfer on JZ contraction was investigated in this study. Uterine transmyometrial embryo transfer resulted in a significant increase in JZ contraction.	JZ contraction increased after transmyometrial embryo transfer, which can interfere with the success of IVF.

### 1.3 Effects of estrogen receptors on fertility

**Figure 1:**
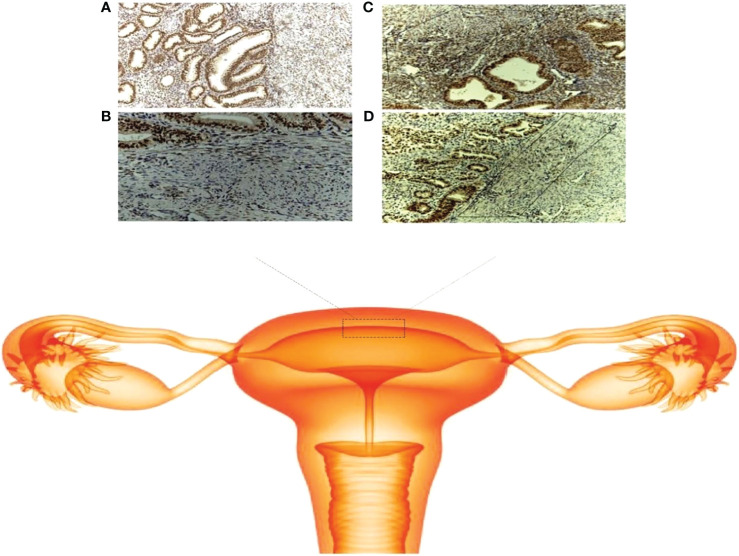
Schematic of the junctional zone. **(A)** ER expression in the adenomyosis group (× 100); **(B)** ER expression in the control group (× 100); **(C)** localization of the JZ in the adenomyosis group (× 100); **(D)** localization of the JZ in the control group (× 100).

Hormones are the primary regulators of female reproductive function, including ovulation, menstruation, embryo implantation, and pregnancy. At present, increasing evidence shows that hormone abnormalities can cause many diseases, which may lead to endocrine disorders among the endometrium, myometrium and cervix, and decidua and trophoblast, thus inducing pregnancy complications. Uterine dysfunction in women with adenomyosis may be due to normal peripheral estradiol levels and local hyper-estrogenism. However, it is still unclear exactly how estrogen influences the uterus. Some previous studies found that there were cyclical changes in estrogen receptor-α (ER-α) expression in the JZ in adenomyosis patients but that high expression was persistent (28) (**Figure 1**). As an estrogen-dependent disease, estrogen mediates JZ dysfunction through the constant high expression of ER, which plays a significant role in the pathogenesis of adenomyosis. Our study also demonstrated that estrogen could increase the intracellular free calcium of the JZ through a membrane receptor-dependent and nongenomic mechanism of action (29). This may lead to an abnormal contraction frequency and the JZ cycle, leading to disease progression and infertility in patients with ADS. However, this is far from enough to address the question. This suggests that, without a uterine specimen, the persistent high-level expression of ER on the JZ may be used as an indicator to assist in diagnosing adenomyosis. Thus, the pathological diagnosis was achieved under conservative treatment.

### 1.4 Effects of oxytocin receptors on fertility

Oxytocin receptors are widely expressed in human uterine epithelial cells and smooth muscle cells, as well as in peritoneal endometriosis and ovarian endometriotic cysts. Adenomyosis is an estrogen-dependent disease; excessive estrogen may increase oxytocin-mediated uterine activity. In adenomyosis, the expression of oxytocin and its receptor increases, which causes spontaneous peristalsis of the myometrium. Because persistent local estrogen excess stimulates oxytocin through ER-α, it further contributes to uterine peristalsis disorder mediated by oxytocin and its receptor in the endometrium, resulting in JZ damage (30, 31). It is recognized that oxytocin can significantly increase the frequency of uterine peristalsis and is one of the most important mediators of uterine contraction, not only during pregnancy but also during the nonpregnant period. Real-time ultrasound has demonstrated that oxytocin-dependent peristaltic motion is limited to the endometrium and JZ in the nonpregnant uterus. Studies have shown that in myometrial smooth muscle cells, OTR expression levels are positively correlated with contraction amplitude (32). The expression pattern of oxytocin receptors in the JZ of adenomyosis patients is disrupted. The expression pattern of OTR is opposite to that in women with a normal JZ, showing that OTR expression in the proliferative isthmus is significantly lower than that in the fundus. The abnormal expression pattern of OTR may lead to the reversal of JZ contraction, thus interfering with sperm transport (18). Miaomaio et al. (33) studied the changes in oxytocin receptors in the JZ for women with endometriosis. They found that patients with endometriosis had higher serum oxytocin levels and higher uterine contraction rates. For the controls, the expression of OTR in the JZ of the proliferative uterus was significantly higher than that in the secretory phase in the cervical area and the uterine floor area. It was speculated that the JZ responded to the positive and negative effects of estrogen and prostaglandin activity, resulting in the synthesis and reduction of oxytocin receptors. OTR expression in the uterine junction of women with endometriosis appeared to have changed significantly and irregularly. Abnormal oxytocin receptor expression in the JZ in women with endometriosis may lead to abnormal uterine contractility, reduced fertility, and dysmenorrhea associated with endometriosis. Oxytocin increases Ca2+ signaling and uterine peristalsis to a greater extent in adenomyosis. These two changes indicate that uterine Ca2+ oscillation and peristalsis dysfunction may be pathogenic factors of adenomyosis and disrupt embryo implantation, leading to a decrease in fertility in adenomyosis (34).

### 1.5 Effect of the JZ on immunity and of local inflammation on fertility

Immune changes significantly impact the occurrence and development of ADS and decidualization of the endometrium. The endometrial lumen epithelium is generally not adhesive, and the receptive phenotype must be acquired transitorily to allow the attachment and invasion of blastocysts. The “implantation window” starts approximately six days after ovulation and lasts 2 to 4 days, which coincides with the decidualization of uterine tissue. Although the term “decidualization” refers primarily to the transformation of endometrial stromal cells into specialized decidual cells, this differentiation process also involves specialized immune cells, including uterine NK (uNK) cells, macrophages, and changes in smooth muscle cells (35). Therefore, immune changes in the JZ also affect women’s fertility. An altered immune response, which might be the primary abnormality in the pathogenesis of adenomyosis, was reported recently. This results in the disturbance of the JZ. This change may promote the invasion of the endometrium into the myometrium through the interface in adenomyosis. This conclusion may also be supported by the high expression of the IL-18 system in eutopic endometrial tissue in the JZ (36). Activation of inflammatory pathways may be associated with immune and vascular dysfunction in the placenta/decidua interaction, leading to obstetric complications such as fetal growth restriction (FGR), preeclampsia (PE), and preterm birth (PTB) (37). During the process of female pregnancy, embryos and trophoblast cells are successfully implanted into the maternal decidua; trophoblast invasion and spiral artery remodeling are essential steps for a successful pregnancy. The structural and functional impairment of the JZ zone is the cause of pregnancy failure. Obstetric complications are the basis of the disease. During pregnancy, notable vascular changes occur first in the endometrium and then in the uterine JZ, and at the same time, trophoblastic invasion causes the decidualization of maternal tissues. The JZ widely represents the inner third of the myometrium and participates in the placenta along with the endometrium. Therefore, the leading site of vascular pathology in pregnancy is not in the placenta or decidua but in the JZ (38). The placenta is characterized by interstitial and endovascular trophoblast cells invading the uterine JZ with changes in spiral arteries. When endovascular trophoblast cells invade and damage the tissue, they fail to reshape the JZ segment of the spiral route, which leads to different degrees of uterine and placental ischemia, oxidative injury, cell death, and necrosis. Defective deep placentation can cause pregnancy complications, such as abortion, in the second trimester, placental abruption, preterm birth, FGR, and preeclampsia. Defective deep placentation in patients with adenomyosis is due to defective remodeling of the spiral arteries (5). During pregnancy, decidual transformation is insufficient, endovascular trophoblast cells are blocked due to arrest at the level of the JZ, and spiral artery access to the muscular bundles is obstructed, explaining the vascular resistance in pPROM and PTB (39). Defective endovascular trophoblast invasion disorder may be secondary to the absence of natural killer cells in the thickened JZ that are involved in the depth of trophoblast invasion (37). The disruption of JZ before pregnancy can impact placental formation and pregnancy outcomes (16). Continued research in this area may provide new methods for diagnosing adenomyosis.

## 2 Role of imaging in the diagnosis and fertility of the junctional zone

The JZ area changes with age, and the structure and function of the JZ in patients with adenomyosis change, which may be the reason for the increased risk of adverse pregnancy outcomes. However, molecular research on the particular location of the JZ region is not extensive at present. The traditional method of diagnosing adenomyosis is pathologically performed after hysterectomy. Recently, with the enhanced comprehension of the JZ, evaluation by means of various modalities, such as imaging technology, to accurately diagnose adenomyosis has become possible, and imaging technology can also be used to examine its structure and function. The imaging evaluation of the JZ before pregnancy may be helpful for the diagnosis of adenomyosis, the identification of infertility factors, and the risk of obstetric complications.

### 2.1 MRI in the diagnosis and fertility of the junctional zone

**Figure 2:**
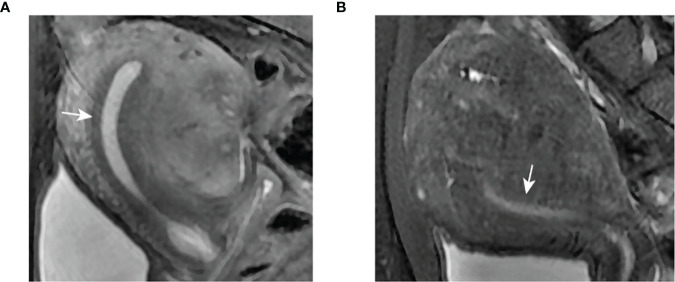
Clear demarcation of JZ appearance on MRI. **(A)** Thickened JZ in a patient with focal adenomyosis. **(B)** Thickened JZ in a patient with diffuse adenomyosis.

In healthy women of reproductive age, the JZ appears as a band of low intensity between the endometrium of high intensity and the outer myometrium of intermediate signal intensity visualized by magnetic resonance imaging (MRI). Many studies have shown that the thickness of the JZ in patients with adenomyosis is significantly different from that on average (**Table 2**). As **Figure 2** shows, a threshold ≥ 12 mm for JZ thickness was described as a critical marker of adenomyosis (46). MRI was reported to have 78% sensitivity and 88% specificity for the diagnosis of adenomyosis because of its ability to visualize the JZ (47). Meanwhile, when coming to less than 8 mm, the thickness could be used as a negative index to exclude the diagnosis of adenomyosis (42). However, according to Tina Tellum et al (48). For the diagnosis of adenomyosis in young women, JZ irregularities might be a more accurate indicator, with a sensitivity of 74% and a specificity of 83%. At the same time, adenomyosis often appears as a local thickening, while diffuse thickening may be physiological, and this characteristic may prevent misjudgment. Although the diagnosis of adenomyosis by means of the JZ is relatively accurate, there are also some flaws in this method. The most obvious problem is that 20% of premenopausal women lack a definable JZ. Fast breath-hold T2-weighted MRI might be helpful to improve performance in measuring the JZ to diagnose uterine adenomyosis (49). MRI can be more accurate in the classification of adenomyosis, although there is no uniform standard for the type of adenomyosis at present. Adenomyosis of the outer myometrium (external adenomyosis), which is not connected with the JZ. The inner myometrium (internal adenomyosis) is characterized by destruction of the JZ accompanied by diffuse growth of the endometrium in the myometrium (50). One study focusing on prepregnancy uterine ultrasound and MR images found that patients with adenomyosis had a 1.84-fold increased risk of spontaneous preterm delivery and a 1.98-fold increased risk of preterm premature rupture of membranes (PPROM) (51). In one study, adenomyosis was diagnosed based on MRI. The IVF/ICSI fresh and frozen-thawed ET were compared according to different classifications. The extrinsic group had a lower abortion rate and a higher live birth rate than the advanced group, which had a higher proportion of miscarriages at or after 12 weeks (52). Classification of adenomyosis by MRI is essential to evaluate patients’ fertility. In focal adenomyosis, sperm transport is mainly hyperperistaltic, while diffuse adenomyosis often shows the loss of sperm transport capacity. Natural conception is complex in this group of women (25). Therefore, MRI examination is essential in infertile patients.

**Table 2:** The different thicknesses of the JZ in MRI for adenomyosis.

Authors	Year	Study
**Reinhold et al., 1996 (** 40)	1996	The authors prospectively studied 119 hysterectomy patients, with an average JZ thickness of 15 mm in patients with adenomyosis and 7.7 mm in normal patients.
**Byun et al., 1999 (** 41)	1999	A retrospective study involved 30 cases of diffuse adenomyosis, with an average JZ thickness of 16 mm.
**Sofic et al., 2016 (** 42)	2016	In a prospective study involving 164 patients with adenomyosis and normal subjects, the average JZ thickness of the former was 14.3 mm and the latter was 5.6 mm.
**Dashottar et al., 2015 (** 43)	2015	This prospective study included 41 patients with diffuse adenomyosis and 19 with focal adenomyosis. The average JZ thickness in each of the two groups was 17 mm.
**Ferrari et al., 2016 (** 44)	2015	This was a prospective trial of 18 patients with a JZ thickness> 12 mm. After 1 year of conservative treatment, 15 of them had a JZ thickness less than 12 mm, indicating that the JZ can be used to evaluate the prognosis of adenomyosis treatment.
**Dueholm et al., 2001 (** 45)	2001	In this study, ultrasound and MRI were performed in 106 premenopausal women undergoing hysterectomy for benign reasons through double-blind trials. It was found that the accuracy of MRI was higher than that of ultrasound, and the difference between the thickest and the thinnest of JZ could improve the accuracy of MRI.

### 2.2 Ultrasound in the diagnosis of the junctional zone

**Figure 3:**
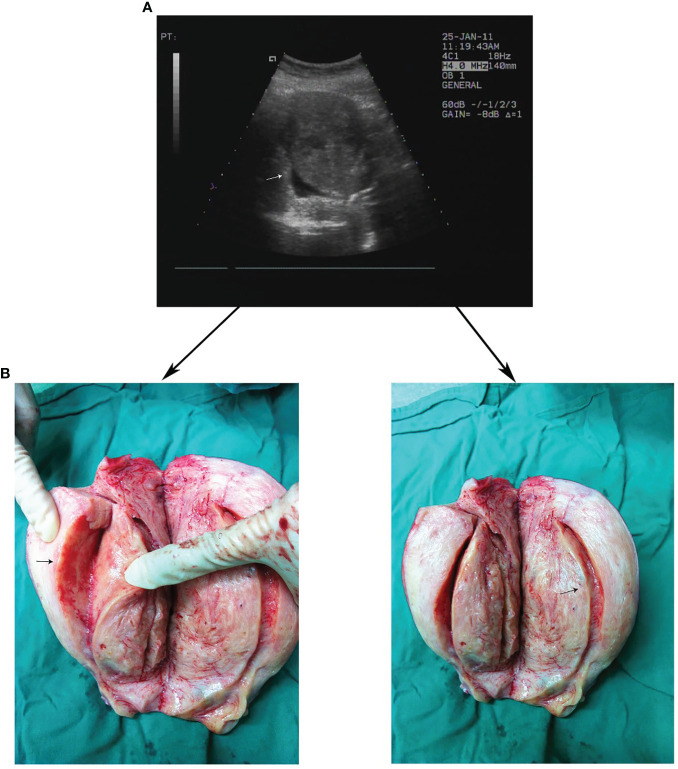
Clear demarcation of JZ appearance on TVUS and gross surgical specimens. **(A)** Thickened JZ in a patient with severe adenomyosis on TVUS. **(B)** Corresponding gross surgical specimens.

Ultrasound research is essential for the comprehension of JZ, and the performance of JZ under ultrasound has a complex relationship with structural organization and biochemical characteristics. Contraction of the JZ under nongravity conditions, which occurs approximately 3-5 times/minute, can be observed by ultrasound (9). The advantages of ultrasound diagnostics are the ease of use, low price, and good exploration quality. With improvements to the technique, ultrasound has gradually become known for its high diagnostic accuracy in detecting the site and position of adenomyosis. As the diagnostic accuracy of 3D-TVUS over the course of JZ thickness maturation has been affirmed and efforts have recently been made to reach an agreement upon radiological criteria in adenomyosis diagnosis, the position of the JZ in the non-invasive diagnosis of adenomyosis has gradually increased (53) (**Table 3**). Reinhold et al. (40). showed that 2D-TVUS and MRI had the same accuracy in diagnosing adenomyosis. With the introduction of 3D-TVUS, high-frequency probes, and more advanced modes, JZ becomes easier to see in the US. 3D-TVUS can be used to evaluate the side and bottom of the JZ and clearly show the endometrium protruding to the myometrium. Unlike traditional ultrasound, 3D-TVUS coronal sectioning can enable a parameter evaluation similar to that of MRI with a higher accuracy, at 84% sensitivity and 84% specificity for the diagnosis of adenomyosis (47). This enables clinicians to evaluate the effect of endometrial ablation and medical therapy through the appearance of JZ alterations, as shown in **Figure 3**. Ultrasound can be used to exclude other complications, enhance the diagnosis of adenomyosis, and give full play to its discrimination of fertility. TVS can also be used to evaluate damage to the JZ in adenomyosis and judge fertility potential. Exacoustos et al. reported that infertility and abortion in adenomyosis at the JZ junction are high (57). Ultrasonic evidence of adenomyosis was found in many infertile women prior to embryo transfer and hurt IVF/ICSI results. Marvelous et al. evaluated the influence of ultrasonic manifestations of adenomyosis on the success rate of IVF. The ultrasonic signs of any adenomyosis are related to the success rate of IVF and the abnormal degree of morphological features indicated by ultrasound (58). Similarly, Dean and others found that the woman’s risk of infertility increased with the increasing number of ultrasound signs of adenomyosis (52). Recent studies have shown that sonographic markers in asymptomatic adenomyosis may not be associated with changes in pregnancy outcomes after transplantation of a single thawed euploid blastocyst. Routine screening for asymptomatic adenomyosis in an unselected population of infertile patients undergoing frozen embryo transfer may not be necessary (59).

**Table 3:** Comparison of the sensitivity and specificity of different methods for detecting the JZ in diagnosing adenomyosis.

Authors	Methods	Sensitivity	Specificity
**C. Exacoustos et al., 2011 (** 8)	2D-TVIS	88%	65%
	3D-TVUS	88%	83%
**M. Bazot et al., 2001 (** 46)	MRI	77.5%	92.5%
	2D-TVUS	65%	97%
**Reinhold et al., 1996 (** 40)	MRI	86%	86%
	2D-TVUS	89%	89%
**Sun et al., 2010 (** 54)	2D-TVUS	62%	68%
**Kepkep et al., 2007 (** 55)	2D-TVUS	46%	82%
**Andres et al., 2018 (** 56)	2D-TVUS	59%	72%
**Andres et al., 2018 (** 45)	TVS	68%	65%
	MRI	70%	86%

## 3 Junctional zone in conservative therapy and reproductive management

**Figure 4:**
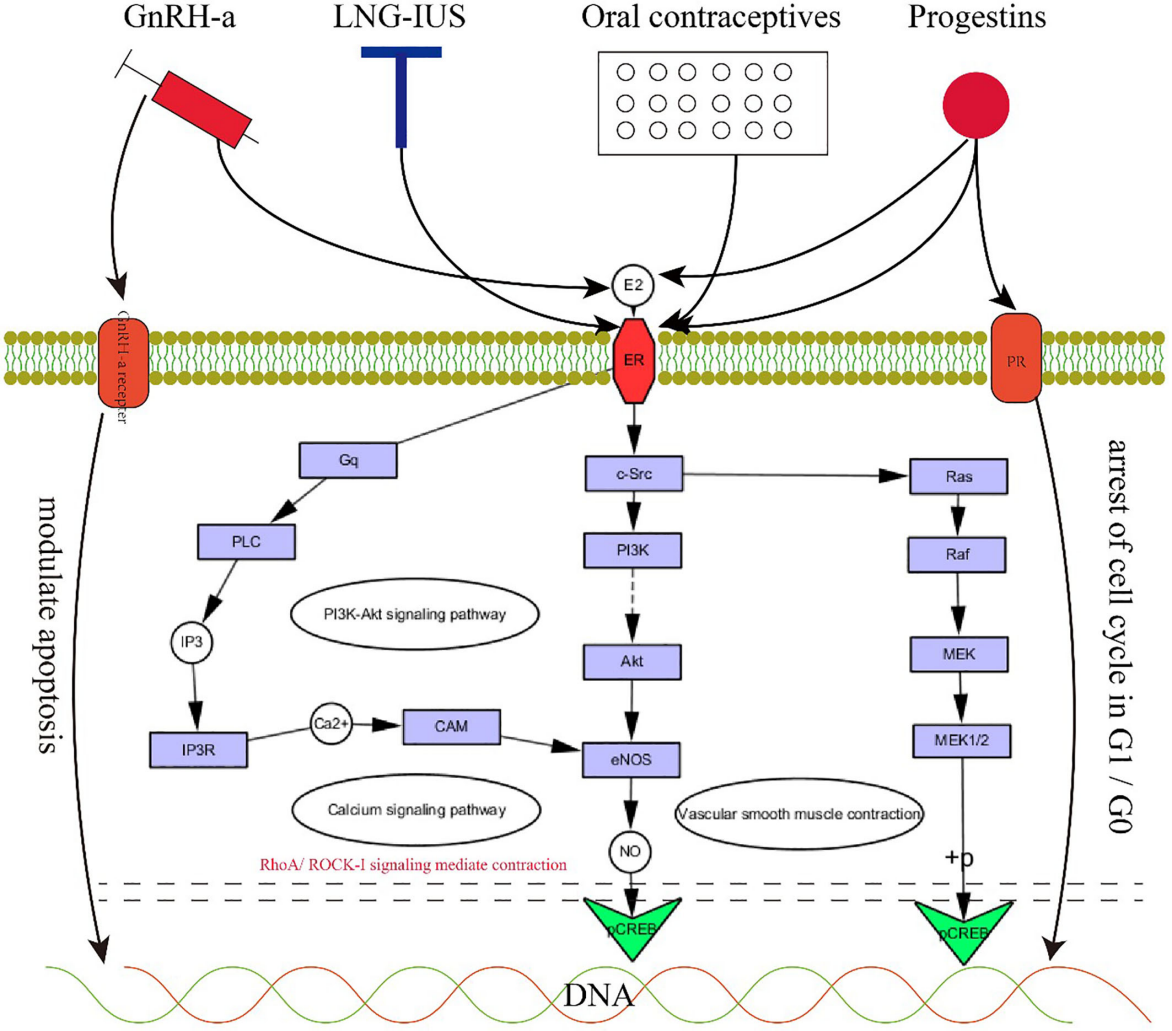
The rationale for medical therapy. 4. GnRH-a, LNG-IUS, oral contraceptives and progestins exert positive effects on JZ by acting on progesterone receptors, estrogen receptors, and GnRH-a receptors to regulate the PI3K-Akt signaling pathway, calcium signaling pathway and vascular smooth muscle contraction.

Although few randomized double-blind clinical studies focusing on medical treatment for adenomyosis have been performed, medical therapy currently shows increasing efficacy in patients requiring control of symptoms or fertility treatments. The rationale for using medical treatment is based on the pathogenetic mechanisms of adenomyosis. Given the critical role of the JZ in adenomyosis, there are a few types of medicine that have a positive effect on the JZ in the treatment of adenomyosis (**Figure 4**). The effectiveness of HIFU on the JZ in the treatment of adenomyosis has also been shown.

### 3.1 GnRH analogues

GnRH-a relieves pain, causes amenorrhea, and significantly reduces menstrual bleeding. For premenopausal women, regardless of the size of the uterus, the use of GnRH-a has achieved excellent clinical results. The rationale for using GnRH analogues for the medical treatment of adenomyosis is the direct antiproliferative effect within the myometrium through actions on the GnRH receptors expressed by adenomyotic lesions with a systemic and local hypoestrogenic effect through a central downregulation and a profound suppression of gonadotropin secretion. GnRH-a can normalize local estrogen metabolism in the eutopic endometrium of women with adenomyosis by decreasing the expression of aromatase cytochrome P450 (60). When GnRH-a suppresses estrogen for a sufficient amount of time, adenomyotic lesions regress. Uterine size decreases, and symptoms are relieved due to the recovery of the structure and function of the JZ in adenomyosis. On the other hand, GnRH-a appears to directly affect endometrial cells by increasing the percentage of apoptotic cells and reducing the release of cytokines such as IL-1β and VEGF, the key molecules in the invagination of the endometrium into the JZ (61). Due to the outstanding efficacy of GnRH-a, it is often used as the first choice for conservative treatment of patients with a large uterus or anemia. The main side effects of GnRH-a are menopausal symptoms caused by low estrogen and the possibility of bone loss with long-term use. There is no consensus on the best treatment for subfertility in adenomyosis. The improved downregulation scheme can improve the clinical pregnancy rate of moderate and severe adenomyosis patients undergoing FTET through the influence of the endometrial inflammatory response and myometrial contractility and its impact on uterine receptivity (62). During IVF/ICSI, the pregnancy outcome of women with adenomyosis who used the ultralong GnRH-a protocol was improved, and the early abortion rate was reduced. In patients with diffuse adenomyosis in particular, the pregnancy and live birth rates have been improved (63). Moreover, an observational cohort study showed that during IVF, patients with adenomyosis had a better clinical pregnancy rate, implantation rate, and live birth rate after treatment with an ultralong GnRH agonist (64). Overall, GnRH-a treatment may benefit the pregnancy outcome of women with adenomyosis, but the exact therapeutic effect still needs more research.

### 3.2 Levonorgestrel-releasing intrauterine system

The levonorgestrel-releasing intrauterine system (LNG-IUS) has been successfully used to treat adenomyosis to reduce menstrual blood loss and pain by reducing the thickness of the myometrial JZ and total thickness of uterine volume. It is recommended as a therapy for patients with hypermenorrhea. Several mechanisms may explain the role of the LNG-IUS in adenomyosis. First, after insertion of the LNG-IUS, the high local concentration of LNG on the endometrium induces endometrial inactivity to estrogen via downregulation of estrogen receptors, resulting in glandular atrophy stromal deciduation on both eutopic and ectopic endometrium that produces a marked reduction in menstrual blood loss. Progestin also acts directly on adenomyotic foci through absorption within the myometrium. In addition, endometrial inactivity can inhibit the activity of aromatase and Cox-2, further decreasing the production of estrogen and prostaglandin, leading to a reduction in JZ hypertrophy and hyperplasia and finally reducing the thickness of the JZ and improving uterine contraction. Bragheto observed a significant decrease of 24.2% in JZ thickness after insertion of the LNG-IUS, and a reduction in pain and abnormal bleeding associated with adenomyosis was also documented. Uterine artery blood flow decreases obviously, along with shrinkage of the uterine volume (65). The LNG-IUS can improve the implantation rates and clinical pregnancy rates of women with adenomyosis who receive IVF. The odds ratio (OR) of ongoing pregnancy increases significantly with the use of the LNG-IUS (66).

### 3.3 Oral contraceptives

The rationale for using oral contraceptives (OCs) in adenomyosis is related to the induced decidualization and subsequent atrophy of the endometrium, reducing pain and AUB. Some studies have reported a significantly thinner JZ in the posterior uterine wall in women with adenomyosis who were treated with OCs compared with women who were not (67, 68). Additionally, a MRI study of nulliparous women showed that the JZ thickness is significantly affected by hormonal contraception (69). Moreover, according to the study by Antoine et al. (15), compared to that of nonusers, the front and back walls of the middle body and fundus of the contraceptive users had significantly thinner connection areas.

### 3.4 Progestins

Progestins are used to treat adenomyosis by inhibiting the secretion of pituitary gonadotropin. It leads to an aperiodic state of low estrogen, giving rise to endometrial decidualization and pseudopregnancy, which appear as amenorrhea. Recent studies showed a correlation between serum progesterone levels and the apparent diffusion coefficient (ADC) of the JZ, which could assess the extent of myometrial invasion of the endometrium (70).

Progestins can relieve dysmenorrhea and reduce menstrual bleeding. However, it is rarely used as a clinical prescription for adenomyosis therapy. Dienogest listed in Japan and Europe has beneficial therapeutic effects. Dienogest is the world’s first specific prescription drug for adenomyosis, and a phase III clinical trial of dienogest was completed in Japan in 2017. This trial demonstrated the good performance of dienogest in relieving dysmenorrhea symptoms of adenomyosis (71). Dienogest can inhibit the proliferation of ectopic endometrial stromal cells by arresting the cell cycle in G1/G0, and P can control E2-induced cell proliferation and increase p27 protein expression in endometrial glandular cells (72). Kazuaki Neriishi et al. (73) conducted a retrospective cohort study to make long-term observations of dienogest. The main side effect is irregular uterine bleeding, which causes 20% of people to stop taking the drug.

### 3.5 Anti-platelet treatment

In addition to the abovementioned changes in the JZ caused by cell cycle and pathway regulation, microtrauma of the myometrium is also an important reason cause of endometrial fragment invasion into the myometrium and eventual adenomyosis. The increase in myofibroblasts in the JZ often indicates the occurrence of microtrauma and simultaneously causes hyperperistalsis and microdehiscences in the JZ, which facilitate the development of adenomyosis (74, 75). Some evidence has shown that platelets might play an important role in adenomyosis pathogenesis because platelets induce epithelial-mesenchymal transition and fibroblast-to-myofibroblast transdifferentiation, ultimately leading to fibrosis (76). A study in a mouse model of adenomyosis demonstrated that antiplatelet treatment (thromboxane A2 synthesis inhibitor) was effective in suppressing myometrial infiltration, improving generalized hyperalgesia, and reducing both uterine hyperactivity and systemic corticosterone levels. In addition, decreased expression of some proteins involved in adenomyosis fibrogenesis was demonstrated, supporting the promising role of antiplatelet therapy in adenomyosis (77). However, to date, no studies have been published or registered on the use of agents targeting platelets.

### 3.6 Focused ultrasound surgery

Focused ultrasound therapy is a minimally invasive treatment for adenomyosis that focuses high-energy ultrasound on focal lesions. The treatment method can be oriented and monitored by ultrasound (HIFU) or MRI (MRgFUS). The advantages of HIFU include its non-invasiveness, lack of radiation and reproducibility. Especially for women with fertility requirements, HIFU has more advantages in maintaining the physiological structure of the uterus and preserving the integrity of the uterus. The disadvantage is that the selection criteria for patients are more stringent. This treatment is recommended only for premenopausal women with adenomyosis without suspicious pelvic adhesions, no history of lower abdominal surgery, abdominal wall thickness <5 cm, and lesion diameter between 3-10 cm (78). A retrospective study evaluated the symptoms of dysmenorrhea and menstrual volume in 350 patients with adenomyosis. This trial showed that the ablation rate of HIFU for adenomyosis was above 70% and that the rate of alleviation of dysmenorrhea was over 80%. Two hundred twenty-four of the patients completed a two-year follow-up, and the rate of symptom relief was over 80%. In one trial, the JZ thickness of 18 patients with adenomyosis who underwent MRgFUS was measured before and one year after surgery. Symptoms resolved in 18 patients one year after treatment, and there was no relapse. The JZ thickness was also significantly lower than that before treatment (44). Therefore, the JZ may be used to predict the prognosis of adenomyosis. Compared with traditional laparoscopy, patients treated with HIFU had higher pregnancy and natural conception rates. Laparoscopic lesionectomy may remove a large amount of myometrium and may reduce myometrial volume, leading to a lack of sensitivity to uterine growth during pregnancy and an increased risk of uterine scarring resulting in uterine rupture (79). The postoperative pregnancy rate of diffuse adenomyotic lesions was significantly lower than that of focal adenomyosis (80).

One clinical study showed that the treatment effect of HIFU combined with LNG-IUS was significantly higher than that of HIFU and HIFU combined with GnRH-a from the perspective of dysmenorrhea degree and menstrual volume (81). However, the treatment of this combination therapy for adenomyosis combined with infertility needs further study.

## 4 Conclusion

In summary, according to current research, changes in the junctional zone affect the occurrence and development of adenomyosis and affect the fertility of women of reproductive age. Therefore, it has gradually become the central research link for ADS patients with infertility. Structural and functional defects of the JZ play an integral role in the development of adenomyosis. Although many conservative treatments have effectively alleviated adenomyosis symptoms, current research on the part of medicine in the JZ lacks a deeper understanding of specific molecular mechanisms. There is still a lack of research on reproductive outcomes after drug treatment. In addition, the changes in the JZ have a significant impact on women’s fertility, but there has been no long-term follow-up study for further observation and confirmation.

Moreover, the treatments mentioned above each have unique associated risks and benefits, and most of them have been studied only for short-term use in small study populations. The efficacy of long-term treatment still needs well-conducted randomized controlled trials. Treatment should be tailored to the individual patient’s specific symptoms or unique request; the same sign may have different implications for different women.

## Author contributions

SW and HD designed the study, retrieved the data, and wrote, revised, and reviewed the manuscript. All the authors have read and approved the final manuscript. All authors contributed to the article.
